# Smoking Habits among Italian Adolescents: What Has Changed in the Last Decade?

**DOI:** 10.1155/2014/287139

**Published:** 2014-04-22

**Authors:** Lorena Charrier, Paola Berchialla, Daniela Galeone, Lorenzo Spizzichino, Alberto Borraccino, Patrizia Lemma, Paola Dalmasso, Franco Cavallo

**Affiliations:** ^1^Dipartimento di Scienze della Sanità Pubblica e Pediatriche, Università di Torino, Via Santena 5 bis, 10126 Torino, Italy; ^2^Dipartimento della Sanità Pubblica e dell'Innovazione, Ministero della Salute, Viale Giorgio Ribotta 5, 00144 Roma, Italy

## Abstract

Tobacco use, alcohol abuse, overweight and obesity are risk factors for numerous diseases in Italy as elsewhere. However, children and adolescents are not usually included in official national surveys although it is at this stage of life when unhealthy habits are often established. Italian participation in HBSC and GYTS surveys allows our country to implement standardized surveillance systems providing reliable information on tobacco-related behaviors of this population. Data from three HBSC surveys (2002–2010) show that following the drop in the first half of the decade, prevalence of tobacco use stabilized in the second half. The decline was significant for younger age groups, while prevalence of regular tobacco use remained stable among 15-year-olds. Many adolescents reported being exposed to secondhand smoke, to have at least one parent who smokes, and having seen teachers and students smoking at school. Although the sale of tobacco products to minors is prohibited, the vast majority had no trouble in buying cigarettes. Data from GYTS and HBSC surveys provide a wealth of information about attitudes and behaviors of Italian adolescents with respect to smoking. Despite some progress, sizeable gaps remain in meeting standard recommendations for discouraging smoking initiation and motivating adolescent smokers to quit the habit.

## 1. Introduction

Despite the many reports on the harmful effects smoking has on health, tobacco remains the world's leading preventable cause of death and disability [1, 2]. Effective tobacco-control programs rely on systematic surveillance to monitor trends in tobacco use. The data so far collected through existing surveillance systems suggest that by 2030 there will be more than 8 million tobacco-related deaths every year largely because of the rising smoking rates among youth, particularly among girls, the high risk of uptake of smoking by nonsmokers, increased exposure to secondhand smoke, and hidden or indirect marketing of tobacco products [3].

Tobacco use, alcohol abuse, physical inactivity, overweight, and obesity are all risk factors for numerous diseases in Italy as elsewhere. However, children and adolescents are not usually included in official national surveys (ISTAT, PASSI, ISS/Doxa), although it is precisely at this stage of life when unhealthy habits are most often established. Regarding tobacco use, most adult smokers lit their first cigarette or were already addicted to nicotine before the age of 18 years [4–8]. Because smoking-related health problems are a function of the duration and intensity of use, smoking prevention in adolescents is of critical concern. The longer the uptake of smoking is delayed, the less likely a person is to become addicted. But once addiction occurs, nicotine dependence is extremely difficult to break. In addition, there is evidence to support a close relationship between cigarette smoking and the use of alcohol and marijuana [9, 10]. In spite of the negative consequences of tobacco use, adolescents may have a positive perception of smoking for many reasons: a way to control negative moods, relax, reduce boredom, belong to a group, control weight, especially among girls, and be identified with a certain image of maturity and self-reliance [11].

The factors that contribute to youth smoking are well documented. The behaviors, attitudes, and expectations of parents and peers can influence the smoking patterns of adolescents [12–16]. This makes it important to investigate such variables as peer relationships, parental support, and school environment to study smoking within a broader context and as part of an adolescent's lifestyle rather than look only at smoking prevalence rates. This was the main aim of the two surveys implemented in Italy. The first is the national health behavior in school-aged children (HBSC) survey in which Italy has participated since 2002. It is a multicenter study carried out in collaboration with the World Health Organization (WHO), coordinated by the Universities of Turin, Siena, and Padua, with the aim of collecting data on health-related behaviors in adolescents aged 11, 13, and 15 years. The second is the global youth tobacco survey (GYTS) promoted by the WHO and the US Centers for Disease Control and Prevention (CDC), which specifically investigates tobacco use among students aged 13–15 years, and implemented for the first time in Italy in 2010.

Equally important is monitoring tobacco use through international and standardized surveillance systems that can capture the changes in habits and attitudes of younger age groups. US data from the national youth tobacco survey (NYTS) indicate that during the past decade (2000–2011) both the prevalence of current tobacco use and cigarette smoking experimentation declined among middle- and high-school students but that the overall prevalence did not decrease from 2006 to 2009 or from 2009 to 2011. Similarly, no change in susceptibility to initiate cigarette smoking was observed [17, 18]. These results are consistent with those from the European School Survey Project on Alcohol and Other Drugs (ESPAD) in which Italy has been involved from the very beginning in 1995. The ESPAD questionnaire starts with a small number of question items on cigarette smoking. Trends for the countries with data from all five waves (1995, 1999, 2003, 2007, and 2011) display, for cigarette use in the past 30 days, a decrease between 1999 and 2007 followed by a stabilization in smoking rates until 2011 [19]. Specifically, for the Italian students, the ESPAD data show a fall in lifetime prevalence between 2000 and 2005, while no significant changes can be detected after 2005 [20].

The aim of this study was to verify on the basis of the results of the three national HBSC surveys (2002, 2006, and 2010) and the first GYTS implemented in 2010 whether the Italian data show a decrease in the prevalence of adolescents who tried smoking and of current smokers in the last decade; an additional aim was to evaluate whether these trends can be seen across all the age-groups involved in the two surveys.

## 2. Methods

### 2.1. Participants

The HBSC is an international school-based survey that collects data on adolescents' health and well-being, social environments, and health behaviors. It consists of repeated cross-sectional cluster sampled surveys among 11-, 13- and 15-year-old students in nationally representative samples of approximately 1500 students from each of the three age groups.

The GYTS is a school-based survey designed to enhance the capacity of countries to monitor tobacco use among students aged 13–15 years and to guide the implementation and evaluation of tobacco prevention and control programs. The CDC normally recommends a sample size of 1500 students for countries participating in the GYTS, as this ensures representative estimates with a precision level of ±5%.

Both the HBSC and the GYTS apply standardized sampling methods for selecting schools and classes, questionnaire design, procedures for conducting the survey in the field (self-completion questionnaires administered in the classroom), and data management (http://www.hbsc.org/; http://www.who.int/tobacco/surveillance/gyts/en/) [21–23].

For the Italian GYTS sample, as agreed with the CDC, sampling was not carried out on the basis of the list of all first and second level secondary schools; instead, schools were selected from those previously sampled for the HBSC study. In agreement with the CDC, the idea was that, for economic and organizational reasons, the GYTS would be conducted on a subsample of the HBSC sample, as the reference schools for both surveys were the same (first and second level secondary schools). Accordingly, the CDC sampled the schools, starting from the list of those surveyed for the HBSC study. The classes from the surveyed schools were randomly selected for participation, starting from a comprehensive list of the previously selected schools.

### 2.2. Ethical Aspects

Participation in the surveys was voluntary and compilation of the questionnaires was anonymous.

The Ethics Committee of the Italian National Institute of Health approved the protocols and methods of the surveys implemented in 2010. Protocols and methodology of the HBSC surveys carried out in 2002 and 2006 were approved by the Board of the Italian Ministry of Health and Ministry of Education.

### 2.3. Measures

The HBSC questionnaire includes mandatory question items investigating tobacco consumption: one is related to smoking initiation (“*Have you ever smoked tobacco? -at least one cigarette, cigar or pipe*”) and another investigates the frequency of the habit (“*How often do you smoke tobacco at present*?”) with response options ranging from “*I do not smoke*” to “*every day*.”

The GYTS questionnaire comprises a core set of items that are used by all participating countries and that investigate seven domains: prevalence of tobacco use, knowledge about and attitudes toward smoking, role of the mass media and advertising in encouraging/discouraging tobacco use, accessibility to tobacco products, antitobacco education in school, exposure to secondhand smoke, and smoking cessation.

In order to make comparisons with the HBSC survey regarding the prevalence of tobacco use, we analyzed the responses to the following questions: “*Have you ever tried or experimented with cigarette smoking, even one or two puffs*?” to evaluate first experimentation with tobacco and “*During the past 30 days, on how many days did you smoke cigarettes?*” (response options ranging from “*0 days*” to “*all 30 days*”) in order to assess the prevalence and frequency of a regular habit.

### 2.4. Statistical Analysis

Weighted prevalence estimates and 95% confidence intervals (95% CI) were computed for each age group and gender. The significance level was set at *P* = 0.05. All analyses were performed using the Stata 12 statistics package. 

*HBSC.* The 2010 HBSC database was linked to the 2002 and 2006 databases for calculating the trend analyses. All analyses are design-adjusted to take account of the effect of the complex survey design (stratification, clustering, and weighting) on the precision of the estimates.

Trying smoking and daily cigarette use trends over time were evaluated using logistic regression analyses. Having tried smoking (yes/no) and daily cigarette use (yes/no) were used, respectively, as the dependent variable and survey year and Family Affluence Scale (FAS), a four-item measure of family wealth used in the HBSC as a measure of socioeconomic status (SES), as independent variables [24]. Analyses were stratified by age group and gender and simultaneously assessed for linear and quadratic (nonlinear but significant trend over time, depicted by a curve with one bend) trends. The significance of the trends was tested from the *P* value of the slope coefficient *β* from the logistic fitting process.

*GYTS.* The GYTS data are weighted to adjust for sample selection (school and class levels), nonresponse (school, class, and student levels), and poststratification of the sample population relative to the grade and sex distribution in the total population.

## 3. Results and Discussion

*HBSC.* Data were available for 4811 students from the 2010 survey: 50.1% males and 49.9% females. Survey population breakdown: 33% 11-year-olds, 34.9% 13-year-olds, and the remaining 32.1% 15-year-olds. The distribution by sex and age was similar in the 2002 and 2006 surveys.

Trend analyses for the three Italian surveys (2002, 2006, 2010) refer to 13,088 adolescents. 

*GYTS*. Out of the 1854 response sheets that were completed and returned, we report on the 1587 sheets completed by students belonging to the survey target age groups (those who stated being 13, 14, or 15 years of age when they completed the questionnaire). Males made up 47.6% of the sample (52.4% females). The population breakdown by age group was: 35.1% 13-year-olds, 32.3% 14-year-olds, and the remaining 32.6% 15-year-olds.

Table 1 shows the results of the two surveys implemented in 2010, referring to the prevalence of adolescents who stated they tried smoking and those who reported smoking every day. These two question items, and their similar modalities, allow in this case for a direct comparison between the HBSC and the GYTS data.

The data reported in Table 1 show that, for both items, the results from the 2010 GYTS and HBSC surveys are substantially coherent and show no statistically significant differences in the results of the analysis of the two age groups (13- and 15-year-olds). More marked differences, statistically significant for the females who reported having experimented with smoking, emerge between the two surveys when comparing data by gender (excluding in this case the HBSC data for the 11-year-olds as they are not compatible with the GYTS data). A possible explanation for the differences resides in the fact that the comparison is made between two age groups (13- and 15-year-olds) for the HBSC versus three age groups (13-, 14-, and 15-year-olds) for the GYTS, where the analyses by age group showed precisely that in the move from 13 to 14 years of age there was a significant rise in the prevalence of adolescents who reported having experimented with smoking.

**Figure 1:**
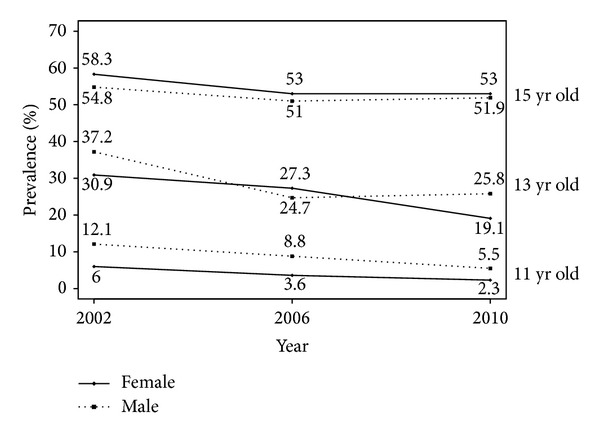
Prevalence (%) of students who reported having tried smoking. Trends stratified by age group and gender (HBSC 2002–2006-2010). Results from logistic regression analyses controlling for FAS. 11 yr old: statistically significant linear trend (*P* < 0.05) for both males and females. 13 yr old: statistically significant linear trend (*P* < 0.05) for females and square trend for males (*P* = 0.02).

Figure 1 illustrates the time trends for all three age groups and both sexes with regard to first experimentation with smoking (prevalence of early experimentation with smoking). The 2002–2010 HBSC survey data show a significant reduction over time of the prevalence of 11- and 13-year-olds of both sexes who reported they had experimented with smoking. No statistically significant difference emerged for the 15-year-olds of both sexes: following a decline between 2002 and 2006, the prevalence then stabilized in 2010. All surveys showed an inversion in the prevalence among males and females, with more females experimenting with smoking with increasing age, though the difference between the sexes was not statistically significant.

The international HBSC protocol uses the proportion of adolescents who report smoking at least once a week to measure smoking frequency rates and to identify the proportion of those who progress from experimentation to regular tobacco use.  Table 2 reports the data from the three HBSC surveys stratified by age group (13- and 15-year-olds) and sex.

No substantial changes in smoking habits among the 15-year-olds emerge over time. The prevalence remains fairly stable and is nearly always higher among the females, confirming the trend seen for first experimentation with smoking. A downward trend is evident among the 13-year-olds of both sexes, which was statistically significant for the males but not the females.

Trend analysis of the HBSC data for adolescents who reported smoking every day was performed only for the 15-year age group. No significant changes across the three surveys can be noted: the prevalence of daily smokers of both sexes remains about 15% (95% CI: 13.02–16.71) (data not shown).

Our findings from the 2010 HBSC and GYTS are congruent in documenting that in Italy, as in many other parts of the world, tobacco use by young people remains a serious problem: by age of 15 years, over 50% have already experimented with smoking and nearly 15% are daily smokers. Furthermore, the GYTS data on susceptibility to initiate smoking within 1 year among those who have not yet started indicate that 35.4% of the males and 46.6% of the females fall into the susceptible category. (By analyzing the responses to the items “If one of your best friends offered you a cigarette, would you smoke it? ”and “At any time during the next 12 months do you think you will smoke a cigarette?” only from among those students who stated they had never experimented with tobacco, the GYTS derives the finding related to the susceptibility of the interviewees to start smoking. Under the protocol, the “susceptible” category was defined as those who, never having smoked, gave responses other than “definitely not” to both these items.)

The HBSC data on the trend for the past decade are substantially in line with published data that show a drop in the prevalence of experimentation with smoking and regular tobacco use among adolescents. The estimated prevalence stabilized in the second half of the decade, however [17–20]. In detail, the Italian data show a significant drop in first experimentation only for the younger age groups (11- and 13-year-olds), with a less pronounced decline, or stabilization, between 2006 and 2010. Similar trends have been observed in other studies, some of which also carried out on HBSC data: a German paper analyzing data collected in the same time frame as ours (2002–2010) shows a strong decrease in the use of psychoactive substances (tobacco, alcohol, and cannabis) by adolescents, but also a clear flattening of the decrease from 2006 to 2010 [25]. A Dutch study published in 2010 demonstrates a clear decline in ever and current smoking between 1996 and 2005 among adolescents; the same results were highlighted in the Dutch HBSC data, showing a continuous decrease in the lifetime prevalence of smoking among Dutch adolescents from 1996 to 2005 [26].

One possible explanation for the Italian trend is the implementation of various interventions during the early years of the decade, among which were restrictions on access by minors to tobacco products and the ban on tobacco product advertising in the media. Between 2003 and 2004, the Italian government implemented European directives on banning tobacco product advertising and misleading consumer information that some tobacco products were less harmful than others. As an additional deterrent to the purchase of tobacco products by minors (<16 years of age), starting in 2004, cigarette vending machines can be operated only during nighttime hours. Since 2007, the machines must contain an electronic device to verify the age of the buyer. But it was with the enactment of Law 3/2003 that the public began to realize the harmful effects of smoking on the health of smokers and those exposed to secondhand smoke. As of January 2005, smoking is prohibited in indoor public places, including bars and restaurants, as well as public and private workplaces. Already before the law went into effect, largely through the print media, greater attention directed toward smoking-related health problems helped to grow awareness and perception of the risks associated with smoking and influence adolescents' attitudes toward smoking.

These interventions appear to have been effective in reducing first experimentation with smoking among the younger age groups but not among the 15-year-olds and to have failed to reduce the prevalence of current smokers or daily smokers which has remained substantially unchanged over time. From this we may infer that antismoking policies seem to have delayed initiation among adolescents rather than reducing uptake by the younger age groups and to have failed to reach young smokers already addicted to the habit.

Another plausible explanation for the stabilization of smoking prevalence after 2006 is that no new antismoking laws were enacted and that attention to smoking risks waned following the success of Law 3/2003. Many Italians recognized the benefits of antismoking legislation: more than 90% were moderately to strongly in favor of smoke-free areas in public places and about 87% supported the ban in workplaces. The data demonstrate its short-term effect on cigarette sales (down 8.9% between 2004 and 2005) and on consumption, with a greater decline among women and young people [27, 28].

In this perspective, the GYTS data provide useful information for shaping an effective public health response. The data depict the current situation of adolescents and their attitudes toward smoking, in which there are sizeable gaps in meeting recommendations for reducing the prevalence of smoking and raising awareness among adolescents about the known health risks associated with smoking.

Youth protection laws and antismoking legislation in general (prohibition of selling tobacco products to minors less than 16 years of age —Royal Decree 2316/1934, amended in 2012 with harsher penalties imposed on retailers, and the legal age of sale of tobacco products raised to 18 years, and ban on smoking in public places and schools) are sometimes disregarded and lessons to adolescents by adults or other reference persons about smoking often fall short of expected goals. There is cause for concern when, on the one hand, the data indicate that over half of 14-year-olds have already experimented with smoking and that by age of 15 years over 10% are daily smokers and have no desire to quit the habit, yet, on the other hand, parents and teachers often smoke at home or school: 46% of the students involved in the GYTS reported having at least one parent who smokes. When stratified by smoking status, a significant association emerged (*P* < 0.01) between smoker or nonsmoker status and having a parent who smokes: about 42% of the students who do not smoke and 56.6% of current smokers have at least one parent who does smoke. Nevertheless, 78% of responders, with no differences between the smoker and the nonsmoker groups, responded that the harmful effects of smoking had been discussed in the family. Moreover, smoking inside school buildings is prohibited by law (Law no. 584/1975 and Law no. 3/2003) and ensuring that schools are smoke-free environments is one of the policies the literature considers effective to make smoking less acceptable in everyday life and to effectively discourage students from starting the habit [29–32]. Thanks to the results obtained through surveillance studies on adolescents conducted to date, new legislation has been recently enacted, including the increase in the legal age of sale of tobacco products mentioned above and, in mid-2013, the introduction of smoke-free ordinances prohibiting smoking near school grounds. The data collected through the 2010 GYTS show, in fact, that both teachers and students were often seen smoking inside and outside the school building: 44% and about 56% of the responders said they had seen teachers and students, respectively, smoking inside the school building. This happened though about 60% of the students stated that during the past school year they had been taught in class about the dangers of smoking or had discussed in class why people of their age smoke.

In brief, adults who smoke are not in a position to give children lessons about smoking. In addition, the vast majority of adolescent smokers had no trouble in buying cigarettes at a tobacco store: 92% of those who bought cigarettes over the counter were not refused the purchase because of their age.

Media messages about smoking are often contradictory: over 90% of GYTS responders stated having seen/heard health warnings about smoking yet 98% recall having seen show business personalities smoking in a film or a video and 64% recall having seen a cigarette logo during a televised show or sports event. Far fewer than nonsmokers, 28% of smokers reported owning a gadget displaying a cigarette brand and over 14% said they had been offered free cigarettes by a cigarette sales representative.

Incongruencies in behavior and knowledge emerged on analysis of the responses from the adolescents participating in the GYTS, which probably reflect those of their parents and teachers, as well as deciders, and stem from an adolescent's typical desire to challenge rules, appear grown up, and belong to a group. The GYTS data show that the adolescents surveyed are well aware of the unhealthy effects of smoking: 85% of both males and females stated that smoking was definitely harmful and, when combined with those who stated it was probably harmful, the proportion rose to 97%. Significant differences emerged, however, when the responders were stratified by smoking status: 63.6% (95% CI, 57.5–69.7) of current smokers (under the GYTS protocol, they are the students who had smoked at least on one day during the 30 days before participating in the survey: 20.7% overall, 19.4% of the males and 21.6% of the females) stated that smoking was definitely harmful versus 91.3% (95% CI, 89.5–93.0) of nonsmokers. Overall, about 62% of responders stated that secondhand smoking was definitely harmful and, when combined with the 30.7% who stated that it was probably harmful, the proportion rose to 92%. When stratified by smoking status, only 47% of current smokers stated that secondhand smoking was definitely harmful. Although the data reveal that smokers and nonsmokers are aware of the health risks associated with smoking and exposure to smoke, only 28% of current smokers stated that they wanted to quit the habit and 81.4%, irrespective of sex, responded that they felt they could quit whenever they wanted.

## 4. Conclusions

School-based surveys like the HBSC and the GYTS have several limitations. First, as the survey sample base consists of school attendees, the surveys are not representative of all Italian youths aged 11, 13, and 15 (HBSC) and 13–15 years (GYTS). However, in Italy, as in the majority of countries, most young people in these age groups attend schools. Second, the data apply only to the students who were in school on the day the surveys were administered and who completed the questionnaires. In this respect, the response rates were very high (>80%) for both surveys, suggesting that any bias attributable to absence or nonresponse was limited. Third, the data are derived from self-reports of students who might under- or overreport their behaviors or attitudes. We are unable to determine the extent of this bias; however, reliability studies conducted in the United States have indicated good test-retest results for tobacco-related questions (and for items related to substance use in general) which were not so different from the questions we used in our surveys [33].

In addition, the strength of the reliability of our data is corroborated by the fact that the results of the two surveys do not show significant differences in the response to the items compared here (Table 1) and that they provide comparable estimates of the prevalence for first experimentation with smoking and regular tobacco use (daily smokers).

The results of the 2010 surveys, also as compared with the data from previous HBSC surveys, show that even with a weak signal of a decline or delay in first experimentation with smoking among the younger age groups, the need remains to keep awareness high, implement comprehensive prevention and cessation interventions of proven efficacy, and monitor adherence to current rules and regulations [29–32]. Besides adolescents, an important target for intervention is their reference persons, given that adolescents often learn behaviors and attitudes by their example. What appear to be lacking in Italy are key concepts underpinning recommendations for discouraging the uptake of smoking among younger age groups: initiatives and programs in schools and the family and by decision makers in the adoption of behaviors coherent with the rules to be taught to young people, direct involvement of students and school staff in adherence to maintaining a smoke-free school environment, monitoring of compliance by retailers with the prohibition of the sale of tobacco products either over the counter or by vending machines, and compliance with laws prohibiting smoking in public places and, as of mid-2013, also in outdoor areas near school buildings.

In this context, monitoring the behavior of adolescents and the changes in the contexts in which they live through the administration of surveys such as the HBSC and the GYTS represents an opportunity for Italy to step up to the challenges of implementing and evaluating effective antismoking interventions. The 2014 HBSC and GYTS surveys will provide a basis for highlighting and evaluating the extent of possible improvements in the situation, following the enactment of recent legislation, communication campaigns directed specifically at adolescents and promoted by the Ministry of Health in late 2013, and school-based prevention programs delineated in the Regional Prevention Plans and the guidelines on the primary prevention of smoking published in October 2013.

## Figures and Tables

**Table 1:** Comparison between the 2010 HBSC and the GYTS. Percent prevalence (95% CI) stratified by age and gender of students who reported having tried smoking or who smoked every day.

Item	Tried smoking		Smoke every day	
Survey (2010)	HBSC	GYTS	HBSC	GYTS
Questions and answers used for comparison between HBSC and GYTS	“*Have you ever smoked tobacco? (at least one cigarette, cigar or pipe)*” Yes	“*Have you ever tried or experimented with cigarette smoking, even one or two puffs?*” Yes	“*How often do you smoke tobacco at present?*” Every day	“*During the past 30 days, on how many days did you smoke cigarettes?*” All 30 days

Prevalence (95% CI)				
Age (yrs)				
11	4.0 (2.7–5.8)	—	0.3 (0.1–0.7)	—
13^*∧*^	22.5 (19.9–25.4)	29.0 (22.5–36.3)	1.8 (1.2–2.7)	1.6 (0.7–3.7)
14	—	50.3 (41.1–59.5)	—	8.0 (5.5–11.4)
15^*∧*^	52.4 (49.3–55.6)	60.3 (52.7–67.3)	15.8 (13.7–18.1)	12.7 (9.1–17.5)
Gender (11-year-olds surveyed in the HBSC survey not included)				
Male^*∧*^	38.2 (35.1–41.4)	45.1 (39.4–50.9)	8.2 (6.8–9.9)	5.8 (4.0–8.5)
Female^*∧*°^	*35.5 (32.5–38.6)*°	*46.7 (39.6–53.9)*°	8.8 (7.2–10.7)	8.3 (5.4–12.4)

^∧^There were no significant differences between the results of the two surveys for any of the feasible comparisons, except for females for tried smoking analysis°.

**Table 2:** Prevalence (%) of smoking at least once a week. Trends by age group^*∗*^ and gender (HBSC 2002-2006-2010).

HBSC	2002 prevalence (95% CI)	2006 prevalence (95% CI)	2010 prevalence (95% CI)	*P*-value for linear trend
Smoked at least once a week				
13 yr old				
M	*8.6 (6.2; 11.8) *	*6.1 (4.1; 8.9) *	*5.1 (3.7; 7.2) *	*0.037 *
F	6.7 (4.5; 9.7)	5.5 (3.6; 8.2)	4.3 (3.0; 6.0)	0.170
15 yr old				
M	21.3 (17.5; 25.6)	20.0 (16.8; 23.7)	21.9 (18.9; 25.3)	0.957
F	24.8 (21.1; 28.9)	19.6 (16.0; 23.9)	23.1 (19.6; 27.0)	0.637

*11-year-olds not included due to small numbers.
